# Supplementary figures and images for: Immortalization of American miniature horse-derived fibroblast by cell cycle regulator with normal karyotype
Source: PeerJ. 2024 Jan 26;12:e16832. doi: 10.7717/peerj.16832 (PMC10823992; doi:10.7717/peerj.16832)

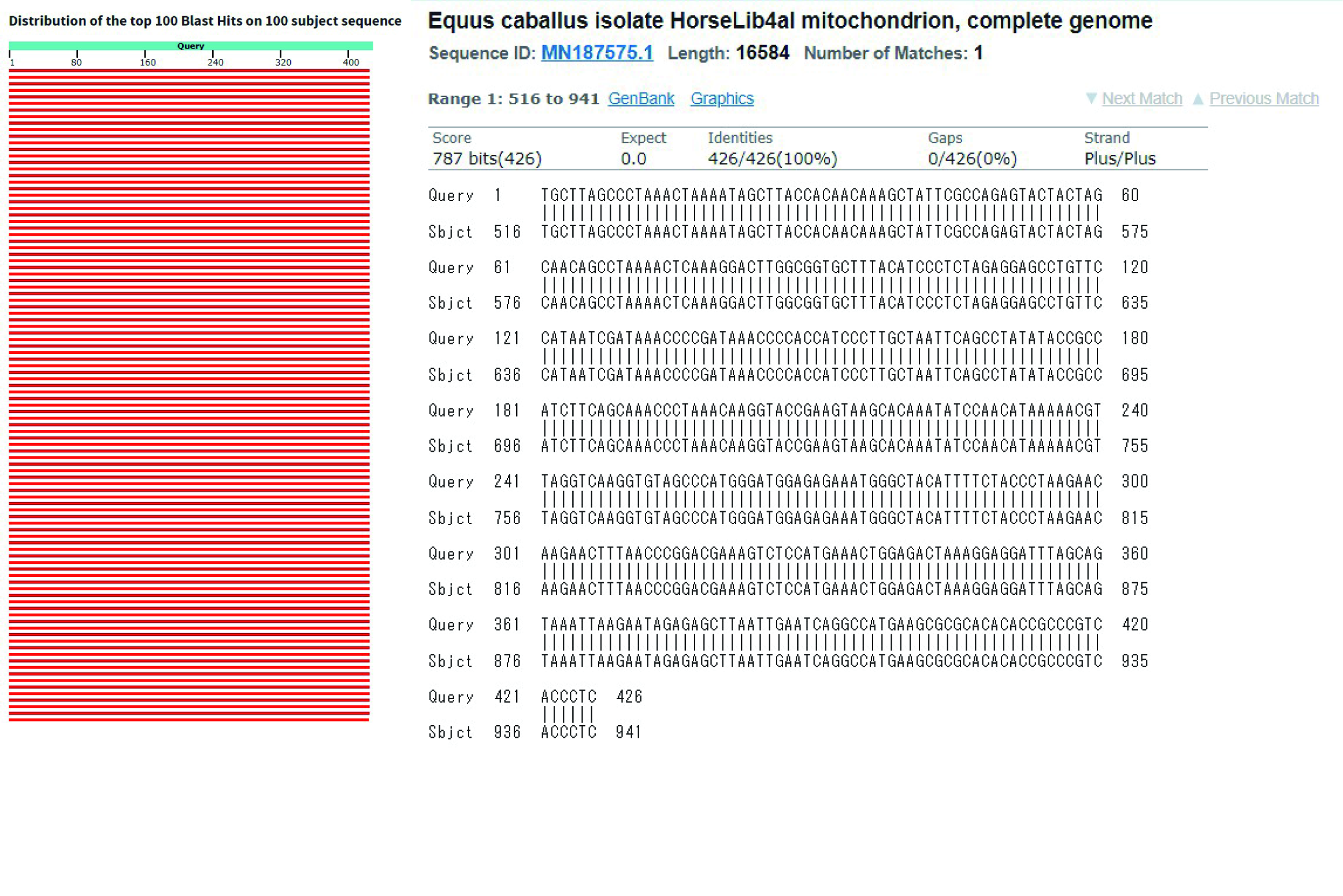

Supplement: Supplemental Information 1 — Although the identity is only 100%, the profiles show that the complete PCR amplicon of the American miniature horse’s mitochondrial 12S rRNA gene only matches the Equus caballus (MN187575.1) mtDNA. [file peerj-12-16832-s001.tif]

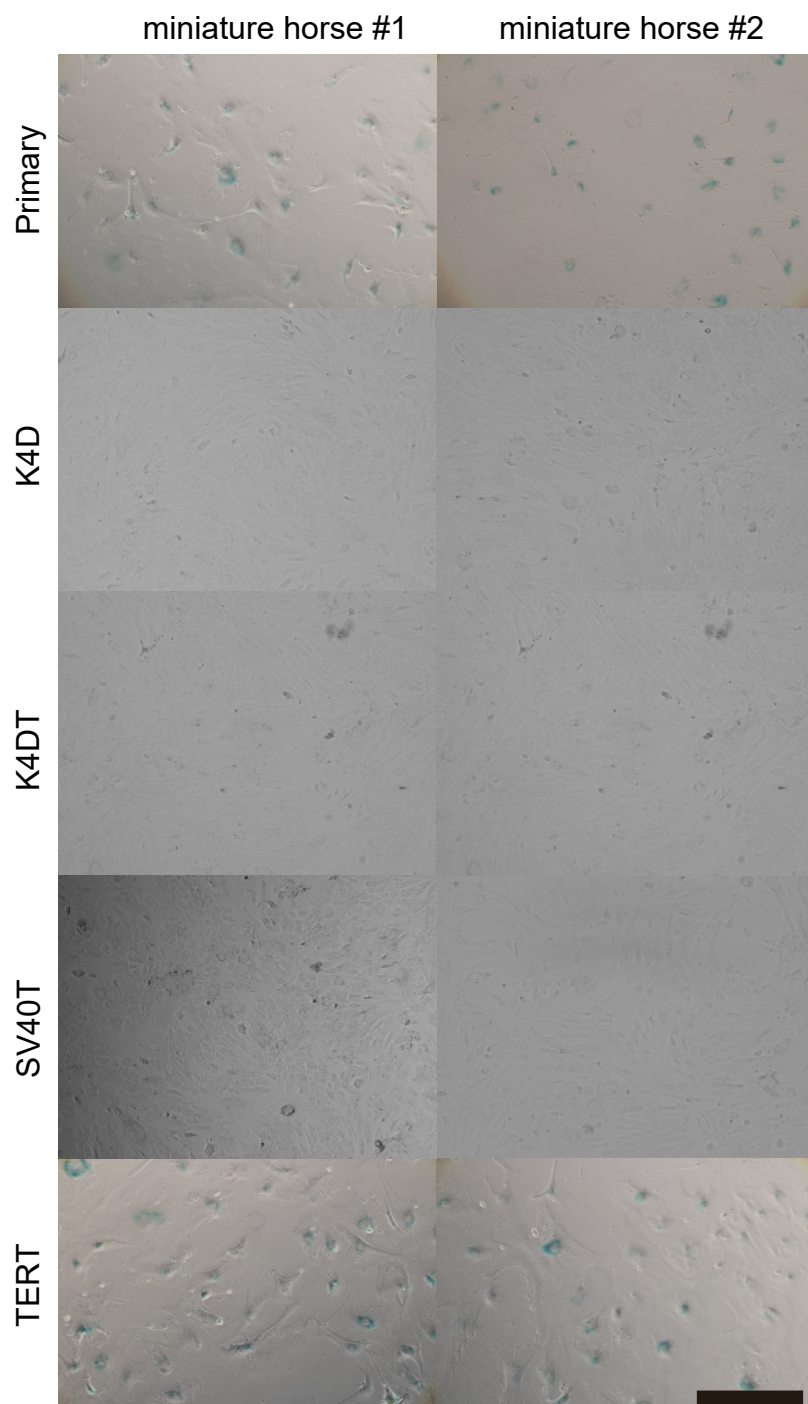

Supplement: Supplemental Information 3 — Each of the cells was stained by SA-beta Gal stain. Scale bars = 50 um. [file peerj-12-16832-s003.pdf]
